# Harnessing ChatGPT for Medical Education: A Narrative Review

**DOI:** 10.7759/cureus.107572

**Published:** 2026-04-23

**Authors:** Biswajit Dey, Himesh Barman, Bishwajeet Saikia, Amitav Sarma

**Affiliations:** 1 Pathology, North Eastern Indira Gandhi Regional Institute of Health and Medical Sciences, Shillong, IND; 2 Paediatrics, North Eastern Indira Gandhi Regional Institute of Health and Medical Sciences, Shillong, IND; 3 Anatomy, North Eastern Indira Gandhi Regional Institute of Health and Medical Sciences, Shillong, IND

**Keywords:** artificial intelligence, chatgpt, digital learning, large language models, medical education

## Abstract

Artificial intelligence (AI), particularly large language models (LLMs) such as ChatGPT, is rapidly transforming medical education by introducing innovative, learner-centered approaches. ChatGPT, a generative AI tool, has demonstrated significant potential in enhancing teaching, learning, assessment, and curriculum design. This narrative review provides a comprehensive overview of the applications, benefits, and limitations of ChatGPT in medical education.

A literature search was conducted in PubMed and Google Scholar for studies published up to 2025, focusing on ChatGPT, AI, and medical education. Relevant peer-reviewed articles, including original studies and reviews, were analyzed and synthesized qualitatively.

ChatGPT serves as a valuable tool for medical educators by facilitating the generation of teaching materials, assisting curriculum development, and supporting assessment and feedback. For learners, it functions as a virtual tutor, promoting self-directed learning through interactive explanations, clinical simulations, and self-assessment tools. Its ability to provide personalized, real-time, and multilingual support makes it particularly relevant in diverse and resource-constrained settings. In the Indian context, ongoing digital education initiatives further enhance the applicability of such technologies, improving accessibility and scalability of learning.

However, several limitations exist, including the risk of inaccurate or misleading information, potential bias, concerns regarding academic integrity, data privacy issues, and over-reliance affecting critical thinking skills.

In conclusion, ChatGPT represents a promising adjunct in medical education, offering opportunities to enhance learning experiences and educational efficiency. Nevertheless, its integration should be guided by appropriate oversight, ethical considerations, and active involvement of educators to ensure safe and effective use.

## Introduction and background

Artificial intelligence (AI) is a branch of computer science focused on creating systems capable of performing tasks that typically require human intelligence [1]. AI mimics human cognitive functions and has taken center stage in modern innovations, largely impacting education and healthcare [1].

Machine learning is a branch of AI that enables systems to learn from data and improve their performance without being hardcoded. In deep learning, a subset of machine learning, neural network architectures are used. These neural networks enable complex data representations, much like the human brain processes patterns [1,2].

Foundation models use deep learning techniques, employing complex neural networks with multiple layers of interconnected nodes. These neural networks, trained on large amounts of data, are adaptable to different tasks [2]. Generative AI, a subset, is designed to create content, including text, images, audio, and more [2]. The key technologies of transformer-based models use attention mechanisms, allowing the model to focus on the most relevant parts of the input, similar to how humans prioritize important information [2]. Large language models (LLMs) are transformer-based AI models that can understand and generate human language, similar to how humans communicate in conversation [3]. A prominent LLM is ChatGPT by OpenAI, known for its conversational abilities [4].

ChatGPT is an abbreviation for “Chat Generative Pre-Trained Transformer” [4]. Since its launch in November 2022 by OpenAI, ChatGPT has undergone extensive testing across multiple disciplines to ensure that it operates naturally and conversationally [5]. Its predecessors were unimodal, i.e., they could only recognize and generate text content. The latest versions are multimodal, accepting and producing text and image inputs and outputs. ChatGPT has performed well across the full range of iterative diagnostic and management reasoning through successive prompting [2]. It is also being widely adopted in medical education [5].

## Review

Materials and methods

This manuscript is a narrative review summarizing current evidence on the applications, benefits, and limitations of ChatGPT in medical education. A comprehensive literature search was conducted using electronic databases, including PubMed and Google Scholar, for studies published until 2025. Keywords used in various combinations included “ChatGPT,” “artificial intelligence,” “medical education,” “large language models,” “generative AI,” “teaching,” “assessment,” “curriculum development,” and “student learning.” Relevant peer-reviewed articles, including systematic reviews, narrative reviews, original research studies, meta-analyses, and perspective papers, were reviewed. Particular emphasis was placed on studies evaluating the role of ChatGPT and LLMs in teaching and learning, curriculum design, assessment strategies, and student engagement in medical education. Additionally, literature addressing ethical considerations, limitations, and challenges associated with AI integration in education was included.

Efforts were made to minimize bias by including studies from diverse designs and sources and by critically evaluating the literature for potential publication bias, selective reporting, and overrepresentation of favorable outcomes related to ChatGPT. The findings were synthesized qualitatively to provide a comprehensive overview of the current evidence, focusing on practical applications, pedagogical implications, and future directions of ChatGPT in medical education.

ChatGPT: the new paradigm in medical education

ChatGPT is considered a new paradigm in medical education due to its ability to respond to complex queries with coherent, contextually relevant answers [6]. In conjunction with traditional teaching methods, ChatGPT can transform medical education.

Traditional medical education relies on a teacher-centered model, which leads to passive learning [7,8]. ChatGPT challenges this approach by facilitating personalized, student-driven learning through adaptive content generation and interactive dialogues [6,7]. ChatGPT is an intelligent tutor that addresses individual needs and provides feedback. As a consequence, it boosts student engagement, enhances medical knowledge, and supports skill development [6]. This makes it especially effective in educational approaches like problem-based learning (PBL), case-based learning (CBL), and team-based learning (TBL) [6].

ChatGPT can serve both medical educators and learners. It acts as an assistant for medical educators and a virtual tutor for medical learners.

Applications of ChatGPT for medical educators

Concerning medical educators, ChatGPT can (a) generate teaching materials, (b) design a curriculum and develop a syllabus, and (c) help in assessment and feedback (Table 1). These roles are described in detail under the respective subheadings below.

**Table 1:** Roles of ChatGPT in medical education for educators Source: This table is original and created by the authors based on references [8-15].

Role	Key Features
Generation of Teaching Material	Customizable educational content
	Support for personalized learning
	Simulation of clinical scenarios
Curriculum Design and Syllabus Development	Alignment with competency-based medical education
	Support for integrated curriculum design
Assessment and Feedback	Generation of assessment questions
	Automated feedback

ChatGPT for Generating Teaching Material

ChatGPT can be a powerful tool for generating high-quality teaching materials tailored to diverse learning needs in medical education [8,9]. It can create clinical case scenarios, multiple-choice questions (MCQs), flashcards, summaries of complex medical topics, and even simulated patient dialogues for use in PBL, CBL, and patient interaction [8,9]. Whether preparing a PowerPoint presentation (PPT), creating a lesson plan, or generating handouts, ChatGPT supports personalized and adaptive learning by adjusting content to students’ knowledge levels, special needs, and curricular goals [8,9]. This saves time and enhances the efficiency of educators while generating relevant resources for students.

ChatGPT creates simulations for early clinical education [10]. These simulations allow students to practice novel aspects of the clinical curriculum, such as forming independent diagnostic and therapeutic impressions throughout an entire patient encounter [10]. Additionally, the simulations adapt to user inputs, replicating real-life scenarios more accurately than pre-made clinical vignettes from question banks. ChatGPT can also provide specific feedback [10]. The added benefit is that ChatGPT can translate educational materials into different languages [11].

ChatGPT for Curriculum Design and Syllabus Development

ChatGPT can play a pivotal role in curriculum design and syllabus development for medical education [12]. It can assist in drafting learning objectives aligned with competency-based medical education (CBME) and suggest suitable teaching and learning methods as per the topics. Additionally, it can help tailor the content according to regulatory guidelines [12,13]. There is a growing intent among medical educators to gravitate toward an integrated curriculum [12]. Regarding syllabus development, ChatGPT can support educators by integrating topics across pre-clinical, para-clinical, and clinical phases [14]. It can generate class routines while offering ideas for vertical and horizontal integration across disciplines to ensure cohesive and comprehensive learning [12,13].

ChatGPT for Assessment and Feedback

ChatGPT can engender high-quality assessment questions, including MCQs, short-answer questions (SAQs), and modified essay questions (MEQs) that harmonize with Bloom’s taxonomy and core knowledge domains [15,16]. To produce precise, high-quality MCQs, it is crucial to use clear and detailed prompts. This approach will significantly enhance the accuracy of the generated content. Medical educators should review these questions thoroughly before using them in exams to ensure accuracy and relevance [17]. ChatGPT can also grade student essays, allowing teachers to spend more time on other aspects of teaching [15].

Applications of ChatGPT for medical learners

With respect to medical students, ChatGPT can (a) tutor in learning and (b) provide self-assessment (Table 2). These roles are described in detail under the corresponding subheadings below.

**Table 2:** Roles of ChatGPT in medical education for learners Source: This table is original and created by the authors based on references [10,15,18-21].

Role	Key Features
Learning Assistant	Immediate explanations for questions
	Information summary
	Collaboration
Self-Assessment and Feedback	Concept checking
	Instant feedback

ChatGPT as Learning Assistant

ChatGPT can be a learning assistant for students, acting as a virtual tutor [15]. ChatGPT may genuinely enhance learning performance by providing immediate explanations to questions, summarizing information, and enabling collaboration [15,18]. This is important for self-paced learning for students, especially slow learners. It provides valuable support for group discussions among students by offering a framework for discussion, personalized guidance, and real-time feedback [19].

ChatGPT for Self-Assessment

ChatGPT can play a supportive role in self-assessment by offering various tools and functionalities, such as generating practice questions, simulating patient cases, and providing feedback on assignments and exam questions [10,20]. It has democratized the process of formative feedback. Provision of personalized and timely feedback is often impractical in large-class settings and is limited by educators’ time. ChatGPT has emerged as a potential solution to this problem. While most medical textbooks and teaching are in English, ChatGPT’s support for multilingual generation has enabled learners from diverse linguistic backgrounds to seek and learn in their languages [21].

Impact of COVID-19 and post-pandemic use of ChatGPT in medical education

The COVID-19 pandemic significantly affected healthcare systems and professionals worldwide [22]. Similar to its impact on patient care, it also disrupted medical teaching and learning [23,24]. Before the COVID-19 pandemic, the proliferation of social media, digital devices, and online resources was already reshaping the landscape of medical education. However, the COVID-19 pandemic has accelerated the integration of new technologies into teaching, learning, and assessment [24]. As we gradually return to normalcy in the post-pandemic phase, ChatGPT compels medical educators and learners to rethink medical education in a technology-transformed world [25]. This shift underscores the need to thoughtfully integrate AI-driven tools like ChatGPT into medical education to enhance learning while maintaining academic rigor.

ChatGPT in medical education from an Indian perspective

India represents one of the largest user bases of ChatGPT globally, with rapid adoption among students for academic support, learning, and examination preparation [26]. The widespread accessibility of such tools has facilitated self-directed learning and improved engagement across diverse educational settings [26]. A nationwide survey of medical teachers in India revealed a cautious yet hopeful outlook toward the integration of LLMs in medical education [27].

AI is being increasingly adopted across various medical specialties in India, supported by government-led digital and health technology initiatives [28]. Government of India initiatives have significantly advanced digital education by promoting accessible, multimodal, and scalable learning solutions, thereby improving educational reach across diverse socio-economic and geographic settings [26]. Building on this digital foundation, LLMs such as ChatGPT have further democratized education, particularly in developing countries, by enabling low-cost access to personalized learning resources, real-time assistance, and multilingual support [29].

Emerging evidence also demonstrates the applicability of ChatGPT in CBME, including its ability to assist in solving clinical and ethics-based case scenarios [30]. In India, where medical education faces challenges such as faculty shortages and increasing academic demands, generative AI offers scalable solutions for content delivery, self-directed learning, and clinical reasoning support [29].

Risks and limitations of ChatGPT in medical education

While ChatGPT has multiple and potentially limitless uses in medical education, it has its risks and limitations (Table 3). Understanding AI is essential for its appropriate use. The extent to which ChatGPT enhances learning depends heavily on the quality of prompts, the instructor’s integration of the model into the learning ecosystem, and the learner’s ability to assess AI-generated content [31]. Crafting clear, effective prompts is crucial in guiding ChatGPT to create questions that align with educational goals. Another limitation of ChatGPT is its susceptibility to “hallucination.” Hallucinations are confident but inaccurate or fabricated information generated by AI models, particularly LLMs. However, an effective solution to this issue is to refine its medical knowledge and augment its input data [32]. Medical educators should be wary of algorithmic bias in ChatGPT. This is important in the context of medical education [20,33].

**Table 3:** Risks and limitations of ChatGPT for medical educators and learners Source: This table is original and created by the authors based on references [20,31-34]. AI: Artificial intelligence

User Groups	Risk and Limitations of ChatGPT
Medical educators	Generation of incorrect information (“Hallucination”)
	Accuracy and accurate assessment depend on the educator’s AI literacy
	Over-reliance may lead to deskilling
	Relying on biased data
	Feedback may lack nuance
Medical students	Over-dependence may reduce critical thinking
	Accuracy and accurate assessment depend on the learner’s AI literacy
	Plagiarism
	Feedback may lack nuance or context awareness
	Privacy concern

For medical education, accountability lies with educators [20]. Over-reliance by educators on ChatGPT may lead to deskilling and overdependence by learners may lead to diminished critical thinking and self-directed learning skills [20]. Without proper guidance, some learners may treat ChatGPT as an answer-generating model rather than a virtual tutor, leading to superficial engagement [20]. Another concern with ChatGPT is academic plagiarism, which may be mitigated through focused education, fostering comprehensive competencies, and implementing diverse evaluation criteria [20]. While ChatGPT can be useful in medical education for providing feedback and assessment, it has limitations in replicating human subtlety and contextual awareness. ChatGPT’s responses may not always account for the specific complexities of individual learners’ situations, personal experiences, or the broader educational context [34]. Data privacy is another concern with ChatGPT. Medical educators and students should exercise caution when handling patients’ data [20].

Future directions

ChatGPT advances medical education by enabling cross-disciplinary collaboration between medicine and AI. This is an exciting time in medical education, where innovations are eagerly anticipated. Innovations being explored include personalized learning pathways tailored to each student's progress and enhanced clinical simulations for immersive training in clinical decision-making and communication skills [35].

While ChatGPT has effectively contributed to advancing education by enriching instructional resources, it remains only a tool for medical educators and students, not a replacement [29]. Consequently, ChatGPT can serve as a bridge between medical educators and learners by translating complex medical concepts into simplified, interactive explanations tailored to the learner’s level. It should be regarded as a valuable adjunct to educators and learners in the medical education setting rather than as a replacement [36].

## Conclusions

ChatGPT is transforming medical education by extending personalized, interactive, and adaptive learning experiences for both medical educators and students. It is helpful for medical educators in generating high-quality teaching materials, developing syllabi aligned with CBME, and formulating assessments. For students, ChatGPT serves as a virtual tutor, improving self-directed learning through explanations, simulations, and aiding self-assessment. However, with opportunities, ChatGPT also presents challenges. These challenges include algorithmic bias, data privacy concerns, the hazard of academic plagiarism, and the potential weakening of critical thinking due to over-reliance. Notwithstanding the challenges, ChatGPT should be perceived as a powerful adjunct tool rather than a replacement for medical educators.
